# Influential factors on disease activity in Crohn’s disease and their Roc curve evaluation: a cross-sectional study

**DOI:** 10.1186/s12876-024-03211-0

**Published:** 2024-04-03

**Authors:** Jing Wang, Shuping Tong, Bingqing Lu

**Affiliations:** The First Affiliated Hospital of Soochow University, Suzhou City, Jiangsu Province China

**Keywords:** Crohn's disease, Disease activity, Dietary inflammation, Physical activity, Cross-sectional survey

## Abstract

**Objective:**

This study aims to investigate the factors influencing disease activity in patients with Crohn’s disease (CD) and provide insights and references for the management and prevention of CD.

**Methods:**

We recruited CD patients who met the inclusion and exclusion criteria and were treated at the First Affiliated Hospital of Soochow University from November 2022 to June 2023. Generalized linear mixed models were used to analyze the factors affecting disease activity in CD patients. Receiver operating characteristic (ROC) curve analysis was employed to assess the predictive value of these factors for disease activity.

**Results:**

A total of 268 CD participants aged 18 to 65 were included in the study, with over 68% of them in remission or experiencing mild disease activity, indicating relatively good disease control. The results of the generalized linear mixed models showed that older age, absence of diabetes, high levels of physical activity, and a low dietary inflammatory index (DII) were protective factors for lower disease activity in CD patients (*p* < 0.05). ROC curve analysis demonstrated that physical activity level, age, and DII all had ROC areas greater than 0.6 in predicting disease activity in CD patients (*p* < 0.05).

**Conclusion:**

The factors influencing the disease activity of CD patients are numerous and should be given attention. CD patients who are younger, have low levels of physical activity, high DII, and have diabetes are at a higher risk of increased disease activity. By reducing or avoiding the mentioned risk factors and leveraging protective factors, it is possible to mitigate the disease activity of CD to some extent.

## Introduction

Crohn’s disease (CD) is a chronic, inflammatory, and disabling gastrointestinal disorder, and its pathogenesis results from the complex interplay of environmental factors, immune system dysregulation, susceptibility genes, and alterations in the host microbiota, ultimately leading to mucosal damage [1]. It has been reported that only about 20% of CD patients experience a slow disease progression, while as many as 50% of CD patients develop complications such as intestinal strictures, fistula formation, and intra-abdominal abscesses within 20 years of diagnosis [2, 3].

In recent years, the application of biologics has brought new hope to the treatment of CD, significantly improving clinical remission rates and mucosal healing rates for patients [4]. However, currently, CD remains incurable, and the treatment principle is to induce disease remission and maintain remission [5]. Therefore, the proper management of CD patients is of paramount importance.

Diet is considered one of the key environmental factors that influence the onset and course of CD as it is directly associated with the regulation of inflammation and immune responses within the body [6]. Various foods, nutrients, and bioactive compounds have distinct immunomodulatory effects [7]. Some dietary components, such as highly refined starches, saturated fats, and trans fatty acids, are associated with pro-inflammatory potential, while others, like fruits and vegetables, appear to reduce the body’s inflammatory levels [8]. Therefore, a diet with a low Dietary Inflammatory Index (DII) may potentially have a positive impact on disease control in CD patients [9].

The course of CD typically includes periods of exacerbation and remission, with inflammatory cells associated with disease activity playing a crucial role within these phases [10]. Most treatment approaches aim to suppress inflammation and block the cascade of pro-inflammatory cytokines [11]. In this context, physical activity and exercise have also garnered significant attention. Research has shown that a lack of physical activity is associated with an increased risk of low-grade systemic inflammation, while regular exercise is linked to an anti-inflammatory state [12]. Exercise enhances the body’s antioxidant response, reduces age-related oxidative stress and pro-inflammatory signaling, promotes skeletal muscle synthetic metabolism, and activates mitochondrial biogenesis pathways [13]. Additionally, exercise helps improve functional performance and overall health by reducing inflammation and oxidative damage signals in vascular tissues, while increasing the availability of antioxidants and nitric oxide [14].

Therefore, in addition to general clinical data such as disease course, type of biologic therapy, and the presence of chronic illnesses, this study specifically focuses on two variables: the dietary inflammatory index and physical activity level. The aim of this study is to explore and analyze the factors influencing disease activity in CD patients, identify modifiable factors, and provide guidance and references for clinical healthcare providers to better assist patients in controlling their disease and improving their quality of life.

## Materials and methods

### Study population

A cross-sectional survey was conducted by recruiting patients with CD who visited the First Affiliated Hospital of Soochow University from November 2022 to June 2023 and met the inclusion and exclusion criteria. Inclusion criteria were as follows: (1) Diagnosed with CD [2, 15] and with a disease duration of more than one year; (2) Adults aged 18 years or older; (3) Undergoing treatment with biologic agents; (4) Ability to communicate effectively; (5) Informed consent and willingness to participate as a collaborator. Exclusion criteria included: (1) Patients with psychiatric disorders or cognitive impairments; (2) Patients with concomitant serious chronic illnesses (such as severe cardiovascular, neurological, renal, pulmonary diseases, etc.), or cancer; (3) Individuals with impaired physical mobility; (4) Pregnancy. After excluding incomplete or invalid questionnaires, a total of 268 CD patients were included in the study. This study was approved by the Ethics Committee of the First Affiliated Hospital of Soochow University, and written informed consent was obtained from all study participants.

### Measurement

#### Clinical data collection

Clinical data for CD patients, including gender, age, BMI, disease duration, medical history, and the presence of chronic illnesses, were collected through the electronic medical record system.

#### Disease activity assessment

The Best Crohn’s Disease Activity Index (Best CDAI) scale [16] was used as a tool commonly employed in clinical practice and research to assess the severity of the disease. It includes eight dimensions: the number of loose stools in a week, the severity of abdominal pain, the physician’s overall assessment of the patient’s condition, the number of extraintestinal manifestations and complications, the use of opiate antidiarrheal drugs, the presence of abdominal masses, a decreased value in hematocrit, and [100 × (1-weight/standard weight)]. The total score is calculated by multiplying the score for each dimension by its respective weight. A score of < 150 indicates remission, 150–220 indicates mild activity, 221–450 indicates moderate activity, and > 450 indicates severe activity.

#### Dietary inflammatory index (DII)

Dietary assessment was conducted using a Food Frequency Questionnaire (FFQ) [17], and the DII was calculated based on individual dietary information and global average intake levels [18]. The calculation method is as follows: DII for a specific dietary component = (daily intake of that dietary component - global average daily intake of that dietary component) / the standard deviation of global average daily intake of that dietary component × the inflammation effect score of that dietary component. To minimize the impact of outliers and right-skewed distributions, the scores are then converted to percentile values, doubled, and subtracted by “1” to achieve a centered symmetric distribution around “0.” The resulting percentile values are multiplied by the total inflammation effect scores of each dietary component to obtain an individual’s food parameter-specific DII score. Finally, the sum of DII scores for all foods is calculated to obtain the overall DII score. A higher DII total score indicates a greater inflammatory potential of the diet. In this study, the DII covered 30 different nutrients, including proteins, fats, carbohydrates, dietary fiber, cholesterol, various vitamins, minerals, and other food components.

#### Physical activity

Physical activity was assessed using the International Physical Activity Questionnaire-Short Version (IPAQ-SV), which was translated into Chinese by Qu et al. [19]. It primarily consists of seven questions to assess the activity level in the past week, including vigorous physical activity, moderate physical activity, walking, and sitting. In this study, the calculation of overall physical activity considered only the energy expenditure for high and moderate-intensity physical activity and walking for study participants. The total energy expenditure was calculated by multiplying the duration of high-intensity physical activity, moderate-intensity physical activity, and weekly walking time (in minutes) by metabolic equivalents (METs) of 8, 4, and 3.3, respectively.

### Statistical analysis

Data entry was conducted using EpiData 3.1 software, and statistical analysis was performed using SPSS 25.0 software. Categorical variables were described using frequencies and proportions. Quantitative data were subjected to normality testing using histograms and the Kolmogorov-Smirnov (K-S) test. Normally distributed continuous data were presented as mean ± standard deviation, while non-normally distributed continuous data were represented as Median (P25, P75). Variable selection was carried out using t-tests, ANOVA analysis, or non-parametric tests as appropriate, and variables with statistical significance were included in the generalized linear mixed model analysis to investigate factors affecting disease activity in CD participants. In this study, disease activity was transformed into a binary categorical variable based on CDAI scores, and therefore, a binary outcome logistic model (Logit model) was employed for analysis. Receiver Operating Characteristic (ROC) curve analysis was used to assess the predictive value of the main influencing factors for disease activity in CD participants. All *p*-values are two-tailed, and the significance level was set at α = 0.05.

## Results

### Basic characteristics of study participants

In total, 268 CD participants who met the inclusion and exclusion criteria were recruited for the study. Their ages ranged from 18 to 65 years, with the majority being young and middle-aged adults. Among them, 75.7% were male, and 75% had no history of intestinal surgery. The majority of patients resided in urban areas, were married, employed, and had no chronic conditions such as hypertension, diabetes, or anemia. Approximately 61.2% of the participants had at least a high school education, indicating a reasonable level of education. Three different biologic agents were used by the participants, including Infliximab, Ustekinumab, and Vedolizumab, with Infliximab being the most commonly used. More than half of the patients had a disease duration of less than or equal to 5 years. The maximum physical activity level was 1674.7 METS, the minimum was 62.2 METS, and the median was 824.2 METS. The average DII was 0.004 ± 0.05, indicating a relatively low dietary inflammatory potential. Please refer to Table 1 for details.

**Table 1:** Characteristics of participants with different levels of disease activity

Characteristics	Remission and mild activity (*n* = 183)	Moderate and Heavy activity (*n* = 85)	x^2^/F	p
Age, y^△^	36(30,43)	28(20,48)	11.838	0.001
**Sex**				
Male	144	59	2.719	0.099
Female	39	26		
**Permanent residence**				
City	123	67	5.492	0.064
Countryside	60	18		
**Marital status**				
Married	129	63	0.792	0.052
Unmarried	54	22		
**Educational level**				
Less than high school degree	73	31	0.286	0.593
High school degree and above	110	54		
**Work situation**				
Retired	21	7	0.651	0.722
Students	25	12		
Working	137	66		
BMI^△^	21.6(19.8,24.0)	20.9(18.9,24.2)	1.783	0.182
**Biological agents**				
Infliximab	114	52	0.044	0.978
Ustekinumab	41	20		
Vedolizumab	28	13		
**Course of disease**				
q5 years	104	58	3.158	0.076
>5 years	79	27		
**Hypertension**				
Yes	23	11	0.007	0.932
No	160	74		
**Diabetes**				
Yes	6	11	9.121	0.003
No	177	74		
**Anemia**				
Yes	5	14	16.631	<0.001
No	178	71		
**History of intestinal surgery**				
Yes	54	14	5.210	0.022
No	129	71		
Physical activity^△^	918.40(748.6,1101.7)	438.6(218.7,756.0)	79.453	<0.001
DII*	-0.36(0.74)	0.78(0.74)	0.229	<0.001

*Note* *Data are expressed in Mean (SD), ^△^Data are expressed in Median (P25,P75)

### Disease activity and its influencing factors in study participants

**Fig. 1:**
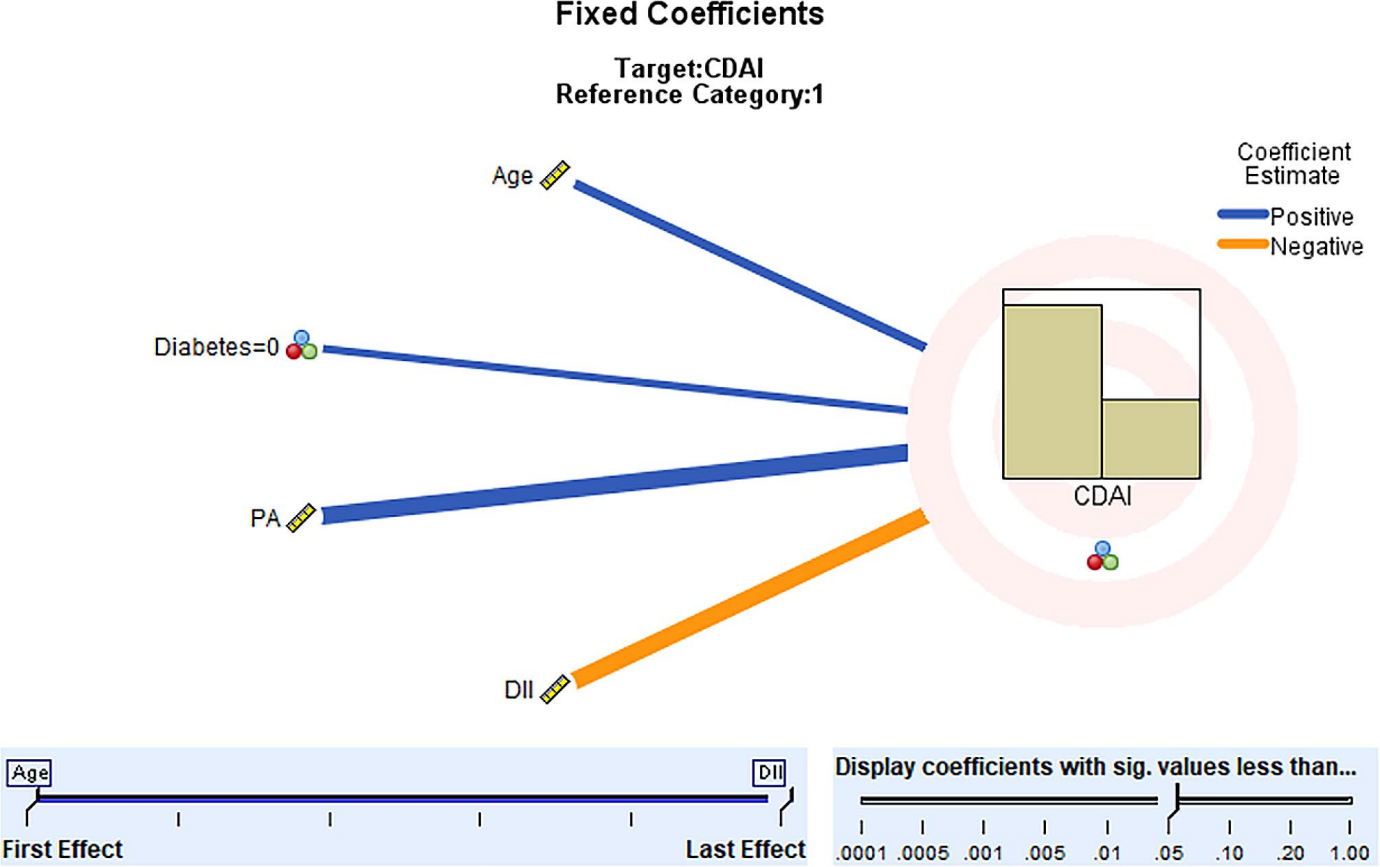
A generalized linear mixed model for factors influencing disease activity in patients with CD. *Note* PA, Physical Activity; DII, Dietary Inflammatory Index; CDAI, Crohn’s Disease Activity Index

Among the 268 recruited CD participants, 183 were in remission or had mild disease activity, indicating that the majority of patients had well-controlled disease conditions. Single-factor analysis revealed that age, the presence of diabetes, anemia, a history of intestinal surgery, physical activity level, and dietary inflammatory index were all statistically significant factors influencing disease activity in CD participants (all *p* < 0.05). Please refer to Table 1. Significant factors identified in the single-factor analysis were included in the generalized linear mixed model (See Table 2 for variable assignments). The results showed that the model with disease activity as the outcome variable was statistically significant (F = 9.250, *p* < 0.001). Older age, the absence of diabetes, higher physical activity levels, and a lower dietary inflammatory index were protective factors for low disease activity in CD patients (all *p* < 0.05). Please see Table 3; Fig. 1.

**Table 2:** Assignment of variables

Variables	Assignment
Disease Activity	CDAI score ≤ 220 = 0;>220 = 1
Age	Original value input
Diabetes	None = 0, Yes = 1
Anemia	None = 0, Yes = 1
History of intestinal surgery	None = 0, Yes = 1
Physical activity	Original value input
DII	Original value input

**Table 3:** A Generalized Linear Mixed Model for Factors Influencing Disease Activity in Patients with CD

Model Term	Coefficient	Std. Error	t-value	p-value	95% Confidence Interval	
					Lower	Upper
Intercept	−5.659	1.438	−3.936	<0.001	−8.49	−2.828
Age	0.056	0.020	2.781	0.006	0.016	0.095
Diabetes = 0	1.559	0.702	2.221	0.027	0.177	2.942
Anemia = 0	1.332	0.798	1.669	0.096	−0.240	2.904
intestinal surgeries = 0	−1.017	0.480	−2.118	0.035	−1.963	−0.071
physical activity	0.004	0.001	4.931	<0.001	0.002	0.006
DII	−1.855	0.355	−5.231	<0.001	−2.553	−1.156

**Fig. 2:**
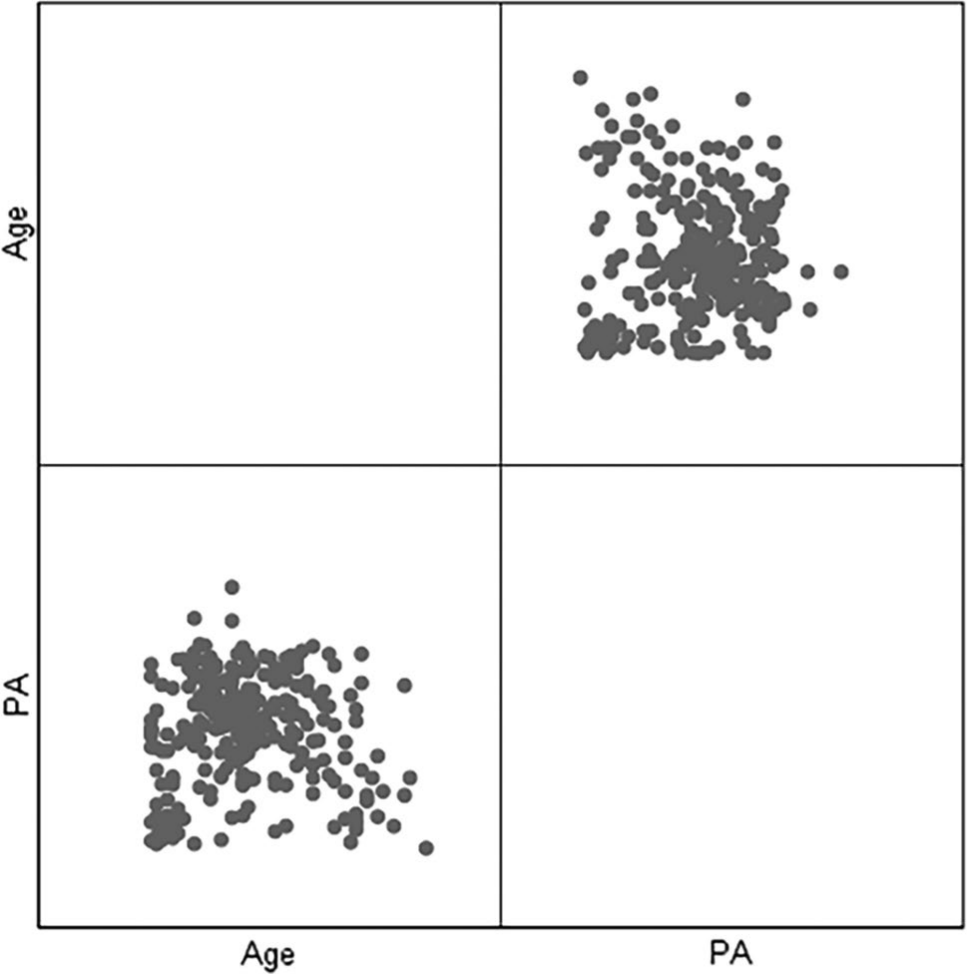
Scattered matrix plot of age and physical activity in CD patients. *Note* PA, Physical Activity

**Fig. 3:**
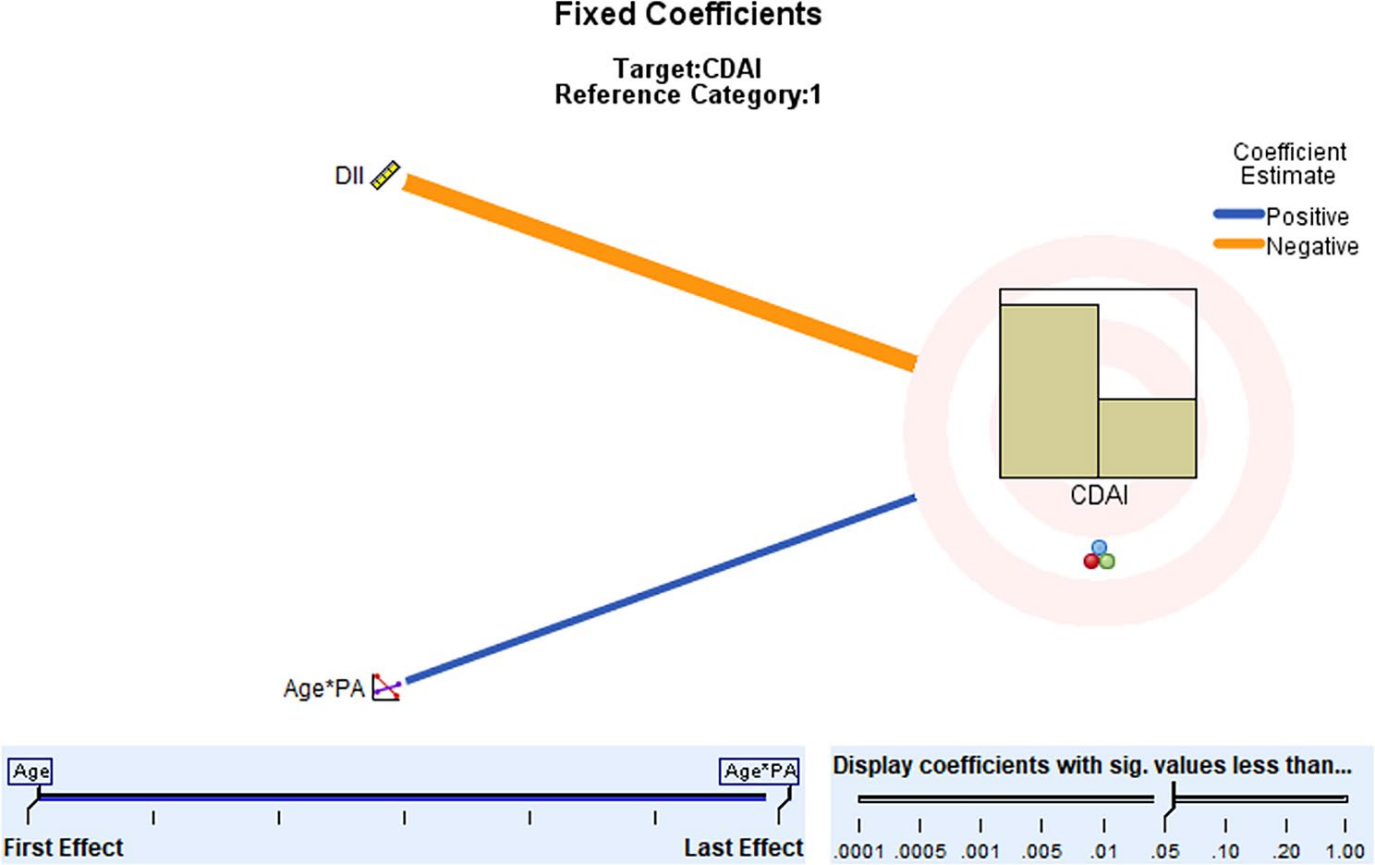
Generalised linear mixed models of factors influencing disease activity in CD patients after adding interaction terms. *Note* DII, Dietary Inflammatory Index; PA, Physical Activity; CDAI, Crohn’s Disease Activity Index; Age*PA Age*PA denotes the interaction of Age and PA

Through scatterplot matrix analysis, it was observed that there might be an interaction between age and physical activity level. Please refer to Fig. 2. An interaction term for age*physical activity level was added to the existing fixed-effects model for further analysis using the generalized linear mixed model. The results indicated that only the dietary inflammatory index and the interaction term age*physical activity level had statistically significant differential effects on disease activity in CD participants. Please see Fig. 3.

To assess the joint effects of factors with statistically significant interaction terms, relative excess risk of interaction (RERI) or interaction contrast ratio (ICR), attributable proportion of interaction (API), Synergy index(S), and their 95% CIs were estimated. ICR = 0, API = 0, or S = 1 was considered indicative of no additive interaction [20]. The results showed that age and physical activity levels had a positive multiplicative effect, with no additive interaction observed. This suggests that age and physical activity level are two independent factors, and their combined effect is not significantly greater than the sum of their individual effects.

### Efficacy of relevant indicators to predict disease activity in CD patients

**Fig. 4:**
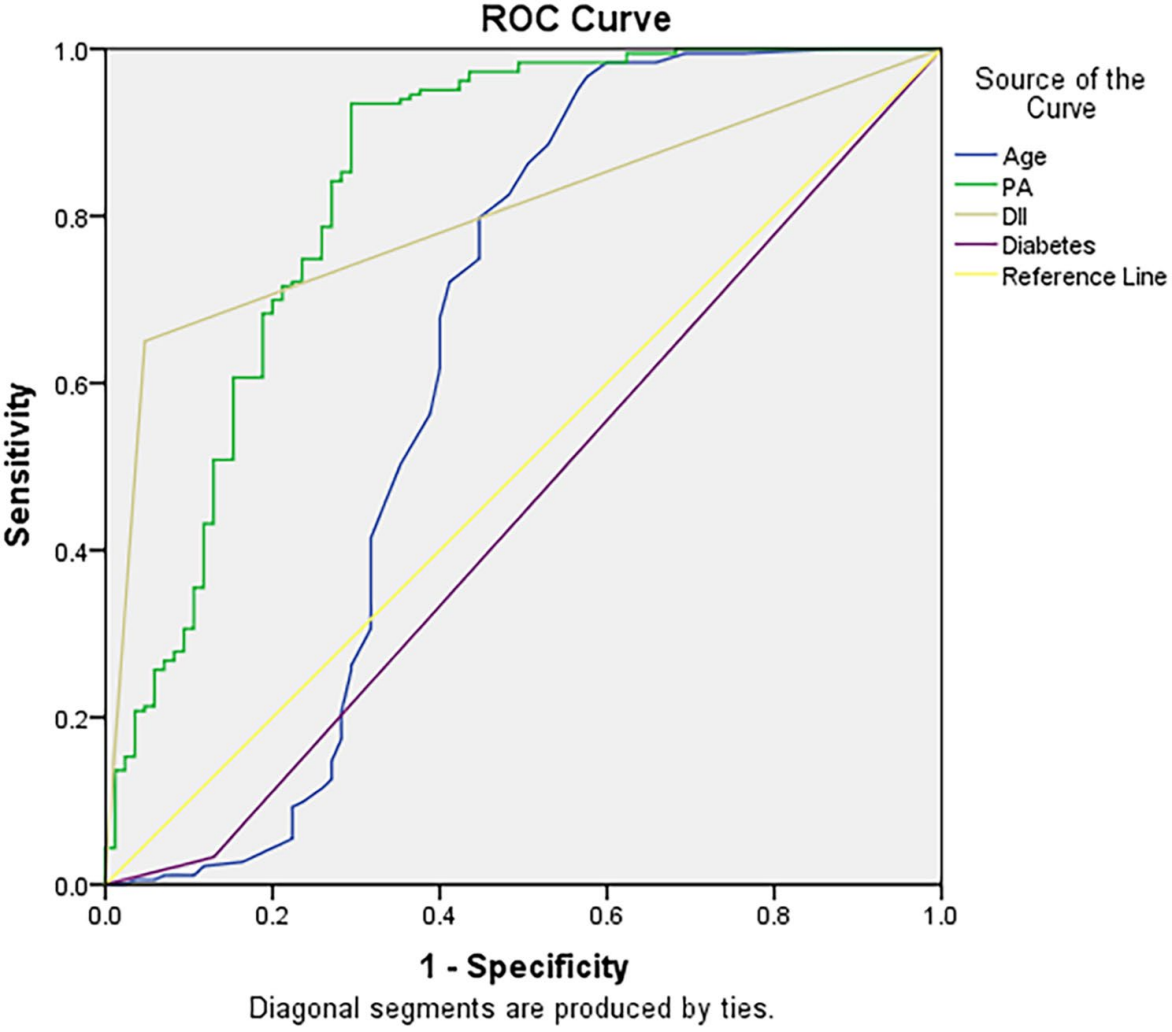
The ROC curve of the main influencing factors predicting disease activity in patients with CD. *Note* PA, Physical Activity; DII, Dietary Inflammatory Index; ROC, Receiver operating characteristic

ROC curves were plotted with disease activity as the dependent variable and the moderately to severely active group as the reference group. The DII was converted into a dichotomous variable, anti-inflammatory diet and pro-inflammatory diet, as smaller values of the dietary inflammation index are better. The results showed that the area under the curve (AUC) for the presence or absence of diabetes was not statistically significant (*p* = 0.203), the AUC for physical activity was 0.838 (95% CI: 0.780–0.897), and the AUC for age was 0.631 (95% CI: 0.541–0.720); the AUC for DII was 0.802 (95% CI: 0.749–0.854); and the sensitivity and specificity were 0.886 and 0.571 respectively. Please refer to Fig. 4.

## Discussion

### Relationship between age and disease activity in CD participants

The results of this study revealed a negative correlation between age and disease activity in CD participants. This finding contradicts previous research results. A study by Liu et al. [21] found no statistically significant differences in CDAI scores among CD patients in different age groups. Similarly, a study by Kamp et al. [22] on the correlation between disease activity and fatigue in inflammatory bowel disease patients did not find an impact of age. Although our study results differ from previous research, this discrepancy may be attributed to various factors, including sample size, study design, and differences in study populations.

It is noteworthy that our study suggests that older CD participants tend to exhibit lower disease activity levels. This could be due to the fact that older patients have more experience and are better at managing their condition, as well as adhering to medical advice. This finding emphasizes the potential importance of age in Crohn’s disease management and suggests that healthcare teams should pay special attention to younger patients to provide more effective treatment and monitoring.

### Relationship between diabetes and disease activity in CD participants

In this study, the results from the generalized linear mixed model showed a significant difference in disease activity in CD participants with and without diabetes (*p* < 0.05). However, contrary to this, the ROC curve analysis results indicated that the predictive efficacy of diabetes status on disease activity in CD participants was low and not statistically significant (*p* > 0.05). This contradictory outcome may partially result from the influence of sample imbalance. There was a significant difference in the number of patients with and without diabetes in this study, which could have led to biases in the calculation of sensitivity and specificity in the ROC curve, thereby affecting the statistical significance of predictive efficacy.

Changes in the gut microbiota play a crucial role in insulin resistance and inflammation [23]. Chronic inflammation can lead to dysbiosis of the gut microbiota, affecting gut immune cells and mucosal permeability, making microbial metabolites more likely to enter the bloodstream, triggering chronic inflammation in the liver and adipose tissue, inducing insulin resistance and high blood sugar [24]. High blood sugar increases the production of oxygen free radicals, exacerbates inflammation, weakens immune function, increases the risk of infection, and forms a vicious cycle [25]. Therefore, future research should aim to expand the sample size to further validate this finding and delve into potential biological mechanisms. This will contribute to providing more reliable scientific basis and guidance for future clinical practice and disease management strategies.

### The importance of physical activity

In our study, we found a significant negative correlation between participants’ level of physical activity and their disease activity. This result suggests that a positive lifestyle and moderate physical exercise may have a beneficial impact on the management of Crohn’s disease, which is consistent with previous research findings [26, 27]. Exercise can reduce the degree of inflammation in CD patients, increase their bone density, muscle mass, and aerobic capacity, improve their fatigue, psychological state, and quality of life [28]. However, compliance with exercise interventions varies significantly among patients with different conditions, and the safety of their application remains to be verified. Clinical controversies regarding intervention methods, treatment intensity, duration, and frequency persist, and there is still a lack of exercise intervention guidelines specifically for CD patients. In summary, based on our study results, we recommend that healthcare teams actively encourage patients to engage in moderate physical exercise to improve the overall health of Crohn’s disease patients.

### Impact of dietary inflammatory index on disease activity in CD participants

Our study results indicate a correlation between the DII and disease activity in CD participants, aligning with previous study results [29, 30]. This suggests that a diet with a low DII may play a role in inflammation and the activity of CD. Prolonged intake of a pro-inflammatory diet can lead to the accumulation of inflammation in the gastrointestinal mucosa, causing damage to gastrointestinal tissues, while long-term consumption of an anti-inflammatory diet can reduce the concentration of inflammatory markers in the body and effectively prevent inflammation in gastrointestinal tissues [31].

Studies [29, 30] also indicate that as the DII increases in CD participants, there is a higher risk of developing sarcopenia, and the IBD symptom score is also elevated. Therefore, CD patients are encouraged to increase their intake of fruits, vegetables and omega-3-fatty acids while minimizing their intake of refined grains, sugary foods, and fatty meats, among other dietary components [32]. However, it is important to note that dietary surveys may be influenced to some extent by patients’ subjective recall and reporting, leading to potential information bias. Future research can delve deeper into how specific dietary factors impact the development and disease activity of Crohn’s disease.

### Other influencing factors

In addition to the factors considered in this study, there are other well-recognized factors worth noting that impact the progression of CD. Previous research suggests a correlation between smoking and the deterioration of CD course, with significantly increased clinical relapse rates and repeat surgery rates in persistent smokers compared to non-smokers or those who quit smoking [33, 34]. Therefore, clinicians should pay special attention to patients’ smoking status and consider this factor comprehensively when formulating treatment plans. Additionally, studies have found that anxiety, depression, and psychological stress are associated with the disease activity in CD patients [35, 36]. Thus, in clinical practice, assessing and providing support for patients’ mental health is crucial. A comprehensive understanding of patients’ physical and mental well-being contributes to addressing the multifaceted impacts of CD, ultimately enhancing the overall effectiveness of treatment.

Moreover, the ECCO consensus [2, 5, 37] and the Chinese guidelines for the diagnosis and treatment of Crohn’s disease [38] also indicate that factors such as younger age at onset, extensive involvement of the intestine, penetrating or narrowing disease phenotype, perianal lesions, and infections may induce or exacerbate CD. In summary, there are numerous influencing factors in the disease activity of CD, and the underlying mechanisms remain unclear. Therefore, considering patients’ lifestyles, mental health, diet, and other potential factors is crucial for effectively managing the disease activity in CD patients. Future research can further explore the complex relationships among these factors to provide more evidence for personalized and comprehensive treatment strategies.

### Limitations of the study

Firstly, this study utilized a cross-sectional research design, and both the DII and IPAQ-SV collected information on participants’ dietary inflammation and physical activity only for the past week. Therefore, causal relationships cannot be determined, and we can only observe correlations. Secondly, the sample size was relatively small, and it was a single-center study, which may limit the generalizability of the results. Future research could consider multi-center collaborations for larger-scale longitudinal studies to establish causal relationships and further elucidate how these factors change over time. Thirdly, the data analysis section of this study was divided into two groups: moderately active and non-moderately active, which enhanced comparability, but the sample sizes of the two groups did differ significantly, which may have introduced a certain degree of bias into the results of the data analysis of this study. Fourthly, this study did not account for some potential influencing factors, such as sleep quality, psychological factors, genetic factors, etc., which could have an impact on disease activity. Therefore, future research should comprehensively consider these factors to better understand the complexity of CD. Lastly, in an effort to minimize the influence of medication on disease activity, this study only included patients treated with biologics. However, this also limits the applicability of the study results, as different types of drug treatments may have varying effects on disease activity. Future research could consider including patients undergoing different treatment modalities to more comprehensively assess the impact of treatment factors on disease activity.

## Conclusion

Despite some research limitations, the findings of this study hold significant implications for the clinical management and health policy development for CD patients. A deeper understanding of these influencing factors can assist healthcare professionals in more comprehensively assessing patients’ disease risks, thus enabling them to take appropriate preventive and treatment measures. This, in turn, can provide patients with more effective medical care and support to enhance their quality of life.

Additionally, future research can delve deeper into exploring the potential biological mechanisms to explain the relationship between the presence of diabetes, dietary inflammatory index, and physical activity levels with disease activity. This may involve detailed investigations into biological mechanisms related to the immune system, inflammatory processes, metabolic pathways, among others, helping uncover how these factors influence the disease activity of CD.

## Data Availability

The datasets generated and/or analyzed during the current study are not publicly available.
